# Light-Intensity-Induced Characterization of Elastic Constants and d33 Piezoelectric Coefficient of PLZT Single Fiber Based Transducers

**DOI:** 10.3390/s130202419

**Published:** 2013-02-12

**Authors:** Lucjan Kozielski, Jiri Erhart, Frank Jörg Clemens

**Affiliations:** 1 Department of Materials Science, University of Silesia, 41-200 Sosnowiec, ul. Snieżna 2, Poland; E-Mail: lucjan.kozielski@us.edu.pl; 2 Technical University of Liberec, Studentská St. 2, CZ-461 17 Liberec 1, Czech Republic; E-Mail: jiri.erhart@tul.cz; 3 Laboratory for High Performance Ceramics, Swiss Federal Laboratories for Materials Science and Technology, EMPA, Überlandstrasse 129, 8600 Duebendorf, Switzerland

**Keywords:** piezoelectric fibers, electro-optical ceramics, extrusion method

## Abstract

Enhanced functionality of electro-optic devices by implementing piezoelectric micro fibers into their construction is proposed. Lanthanum-modified lead zirconate titanate (PLZT) ceramics are known to exhibit high light transparency, desirable electro-optic properties and fast response. In this study PLZT fibers with a diameter of around 300 microns were produced by a thermoplastic processing method and their light-induced impedance and piezoelectric coefficient were investigated at relatively low light intensity (below 50 mW/cm^2^). The authors experimentally proved higher performance of light controlled microfiber transducers in comparison to their bulk form. The advantage of the high surface area to volume ratio is shown to be an excellent technique to design high quality light sensors by using fibrous materials. The UV absorption induced change in elastic constants of 3% and 4% for the piezoelectric coefficient d_33_.

## 1. Introduction

Ferroelectric materials with combined piezoelectric and optical properties are promising materials for electro-optic transducers or piezoelectric transformers with control of output parameter level by optical external stimuli [1]. Such devices could be applicable as light controlled micro electrical components or even as a new energy transfer method in order to supply micro-electromechanical systems (MEMS). The optical supply can be realized using laser illumination, which is very desirable for harsh environments, high-security applications, biological monitoring and arrays of autonomous sensors [2]. It is particularly desirable for MEMS actuators, where the force that can be generated is directly related to the magnitude of supplied voltage that is available. It can be highly important to apply microfiber and voltage gain effective transducers into its structure. Consequently, such new hybrid devices will be able of self-powering by light driven high voltage generation due to the Bulk Photovoltaic Effect (BFE) [3]. At the moment, PLZT is still the most efficient candidate for such applications in spite of the huge progress in lead-free materials. Additionally, PLZT is a photostrictive material, what is a superposition of the photovoltaic and inverse piezoelectric effects [4,5]. Particularly in PLZT, the photostrictive effect is highly effective under light illumination in the near ultraviolet region.

The phase diagram of PLZT indicates a region with pseudocubic symmetry with a slim ferroelectric hysteresis loop (P-E) and no remanent polarization at room temperature, but exhibiting substantial induced polarization under the influence of an electric field. Further addition of La^3+^ element into this solid solution decreases stability of the ferroelectric macro-polar ferroelectric phases and reduces T_C_ with a ratio of 37 degree/mol%.

When the La^3+^ addition reaches 9% it reduces the stable region of the FE rhombohedral phase in a wide range of temperatures so that these compositions are ferroelectric relaxors with characteristic strong frequency dependence on dielectric permittivity [6].

These materials can be used in MEMS devices for light driven high voltage generators [7] to convert optical energy directly into mechanical energy or alternatively, taking advantage of converse BFE. Other interesting BFE applications include photo-driven micromotors, actuators and micro-pumps [8].

More attention has been recently paid to piezoelectric fibers by researchers because they provide excellent flexibility and higher specific strength. Using piezoelectric fiber composites, anisotropy properties can be achieved, which is interesting for new kinds of sensor and actuator applications.

Only a few works have focused on the piezoelectric properties of single fibers because of complicated measurement setups and due to their fragility, also because of a large grain size in comparison to the tiny cross section in the micrometer range [9]. Additionally, metallic electrodes are difficult to precisely deposit on both sides of the fiber. Steinhausen *et al.* [10,11] calculated the properties of ferroelectric fibers by measuring the properties of 1–3 composites by finite element method (FEM) modeling. An alternative method of piezoelectric investigation is a modified atomic force microscopy in piezo response mode called PFM (Piezo Force Microscopy). With this method it is possible to detect the piezoelectric response of thin fibers [12]. With the PFM technique the nano sized tip is effectively used as the top probe of the system, while the bottom of the fiber is fixed to the ground by a conductive silver paste. Hence, it is then possible to determine the piezoelectric response and piezoelectric coefficients can be obtained in the nano-scale range across the fiber surface. The interpretation of micro scale properties is difficult because of the high directional anisotropy effects across the fiber diameter [13]. Clemens *et al.* investigated the large signal properties of PLZT fibers by using a modified dynamic mechanical analyzer (DMA) [14].

The main aim of the present work was the investigation of PLZT fiber which can be used as transducer that combines light induced change in conductivity with excellent piezoelectric properties. Consequently, PLZT fibers that exhibit outstanding electromechanical behavior with optically active properties and transparency were used for this research collaboration. This paper will present the light dependent properties of PLZT fibers suitable for miniaturization of energy transducers.

## 2. Experimental Section

### 2.1. Powder and Fiber Synthesis

In a first step, (Pb_0.93_La_0.07_)(Zr_0.65_Ti_0.35_)O_3_ powder, usually abbreviated as PLZT 7/65/35, was prepared by solid-state reaction of different oxide powders, namely: ZrO_2_ powder (Sigma Aldrich, no. 230693), PbO (Sigma Aldrich, no.211907), La_2_O_3_ (Sigma Aldrich, no. L4000), and TiO_2_ (Sigma Aldrich, no. 248576).

The stoichiometric content of oxide powders, ethanol and 3 mm zirconia balls were mixed in a planetary-mill (RETCH PM400) for 24 h. To compensate the lead loss during sintering, 5 mol% PbO was additionally added.

Subsequently, the slurries were dried and the resulting granulates were uniaxial pressed under 10 MPa pressure into pellets with a diameter of 23 mm. The compacted pellets were calcined in a closed Al_2_O_3_ crucible and PbZrO_3_ powder bed at a temperature of 925 °C for 3 h. After calcination the material was ground for the second time for 24 h in the planetary mill.

After powder processing the particle size and the surface area were measured by laser diffractometer (LS230, Beckman-Coulter Inc.) and BET (SA3100, Beckman-Coulter Inc.). The milled powder was initially coated with stearic acid as a surfactant, the procedure has been described elsewhere [15]. For this step, the appropriate amount of surfactant was firstly dissolved in toluene and afterwards mixed with calcined PLZT powder. The slurry was ball milled for 20 h.

For PLZT green fiber manufacturing, the coated PLZT powder and polyethylene (1700 MN18C Lacqutene PEBD, Elf Atochem S.A.) were compounded in a high-shear mixer (Rheomix 600, Thermo Fisher) at 130 °C until reaching torque equilibrium. The thermoplastic feedstock was extruded through a die with an orifice of 300 micrometers in diameter, using a capillary rheometer (RH7-2, Malvern).

Finally the PLZT fibers where heat treated to remove the polymeric binder and subsequently to densify them at 1,250 °C for 2 h in closed alumina crucible in lead rich atmosphere (PbZrO_3_ + ZrO_2_), further noted as PZ + Z.

### 2.2. Microstructural Investigation of PLZT Fibers

To determine the shrinkage of fibers during sintering, the diameters of green and sintered fibers were measured by using optical microscope (Leica Wild M32). For the microstructure analysis, a fiber was longitudinally embedded into cold resin (Technorit 4000, Heraeus Kulzer GmbH), ground and polished as described by Heiber *et al.* [15].

After preparation, the microstructure was investigated by SEM (Vega Plus 5136MM Tescan). Therefore, the porosity and grain size distribution (after chemical etching) were calculated using the software Digital Micrograph, Gatan (version 3.10.0), and LINCE (version 2.31d), respectively.

### 2.3. XRD Characterization of PLZT Fibers

The phase composition of the sintered fibers was analyzed by X-ray diffraction technique (PANalytical X'Pert Pro Multipurpose Diffractometer) with a Cu Kα source. The 2Θ sweep range was from 5 to 80 degrees, with a step of 0.02. The Rietveld refinement method was used for determination of unit cell sizes applying Scherrer's equation. To evaluate changes of the line profile heights the standard PDF database was utilized.

### 2.4. Light Induced Application Parameters Characterization and Discussion

**Figure 1.**
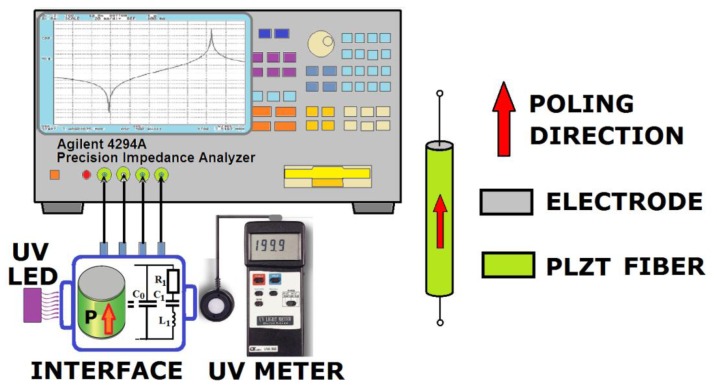
Measurement setup and electrical equivalent circuit of piezoelectric fiber resonator.

An impedance frequency spectrum was measured using Agilent 4294A impedance analyzer in the frequency range from 100 kHz to 10 MHz under dark and illuminated conditions (Figure 1). Temperature in a measurement chamber was controlled prior and after every testing procedure. A thermal bias effect was not observed. Light induced impedance changes near the resonance/antiresonance frequency were generated by UV LED (ELFA 260019 series, 370 nm wavelength, 1 mW optical power) and UV fluorescent lamp (Philips TUV 4W). Electrical equivalent circuit for an ideal piezoelectric element include dynamic branch of capacitor and inductor connected in series. For both afore mentioned elements, capacitance C_1_ and inductance L_1_ define the resonant frequency (Equation (1)):

(1) $$f_{r}=\frac{1}{2\pi \sqrt{L_{1}C_{1}}}$$

The shift of the electrical variables (capacitance and inductance) can be calculated for the piezoelectric fibers in the light and in darkness from the impedance spectrum. Additionally, the recorded changes in impedance modulus and phase level allow determination of piezoelectric coefficient variation, by using the resonance/antiresonance method [16]. Nevertheless, in real ceramics the properties of a piezoelectric resonator has to be studied from an equivalent electrical circuit, which consists of parallel capacitor C_0_ and series of resonant elements L_1_, C_1_, R_1_ (insertion in interface box in Figure 1). Additionally, it was proved, that linear equations of state in real materials transform into non-linear relationships between the elastic and piezoelectric parameters. Precise measurements of piezoelectric resonators show nonlinear change of equivalent circuit elements values, because the resonant frequency depends also on the magnitude of the excitation voltage, induced elastic stress and the actual current value.

Elastic, dielectric and piezoelectric coefficients are obtained from impedance spectrum analysis using a modified resonant method [19]. Free permittivity ε^T^_33_ is determined from a sample's capacitance at 1 kHz. Piezoelectric coefficients and elastic constants can be significantly increased by the light illumination but other studied properties change much less. For characterization of their ferroelectric properties PLZT fibers were cut into 3.5 mm long pieces and placed vertically into an Agilent 16047E sample holder. Both fiber ends were painted with silver conductive paste P-120 ITME to make electrical contact (Figure 1). Subsequently they were poled in silicon oil with 3 kV/mm, 130 °C for 5 min. The illumination was generated by UV LEDs placed at the center of the fiber to provide uniform illumination because the LED light spot is just 3.5 mm in diameter.

## 3. Results and Discussion

### 3.1. Microstructure of PLZT Fibers

After the calcination step, the powder was milled down to the mean grain size 4.06 μm and specific surface area 5.44 m^2^/g. A powder density 8.07 g/ccm was measured with He-pyknometer (AccuPyc 1330, Micromeritics). These three values are important to calculate the right amount of surfactant and material volume inside the torque rheometer.

After sintering, shrinkage 15.5 ± 1.2 % and porosity 0.51 ± 0.29 % were observed. After chemical etching mean grain size 3.7 ± 1.3 μm was calculated. Table 1 gives an overview of calcined powder and the sintered fibers.

**Figure 2.**
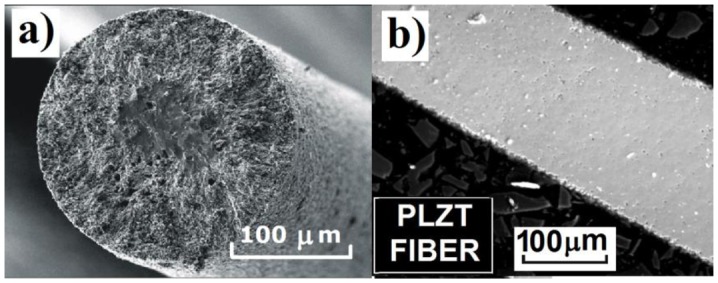
SEM images of the fracture surface of PLZT fiber (**a**) and polished cross section of the investigated fiber (**b**).

Fracture cross section of the obtained fibers was observed using scanning electron microscopy (Figure 2). The morphology inspection did not significantly differ across the diameter and the porosity was homogeneously distributed across the fiber diameter.

### 3.2. Phase Composition of PLZT Fibers

**Figure 3.**
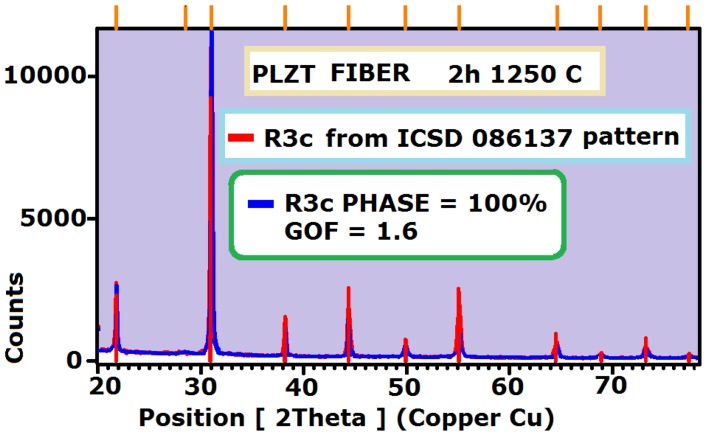
XRD diffraction pattern with the Rietveld approximation of PLZT fibers.

In the obtained diffraction diagrams all indexes connected with the perovskite structure were assigned (Figure 3). Additionally the XRD examination of the fabricated PLZT fibers revealed exclusively stabilized, rhombohedral phase (ICSD collection code: 086137), because of a very low value of goodness fit parameter GOF = 1.6 which evaluates how well the Rietveld model fits the entire data set (a GOF = 1 means the model is as good as possible). As was assumed earlier, an addition of PZ + Z powder creates an atmosphere which prevents lead evaporation during high temperature sintering. Such a protection was successful despite high lead diffusivity, even when the sintering temperature is as high as 1,250 °C [17]. It is worth to emphasize, that the blue peaks at angle 2Θ = 31° in the x-ray diffraction plot were relatively high in comparison to PDF pattern, this fact could be an indication of small texturing created during the extrusion process (Figure 3).

### 3.3. Light Induced Parameter Changes Characterization and Discussion

**Figure 4.**
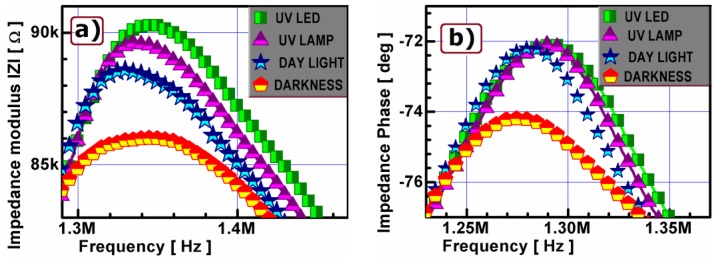
Light conditions influence on PLZT fiber impedance modulus |Z| (**a**) and phase P (**b**) at the antiresonance peak of frequency characteristics.

Figure 4(a,b) shows the complex impedance spectra of PLZT fibers measured near the antiresonant frequency for the different light sources. The changes in light conditions give a distinct effect on impedance maxima shape (Figure 4(a,b)). The impedance modulus and the phase peak are broad in shape for dark and narrow in shape for illuminated fiber. The difference in modulus value is equal to 5 kΩ between UV LED illuminated fiber and not illuminated (dark) one (Figure 4(a)). Under the same conditions a marked change in phase value equal to 3 degrees (Figure 4(b) could be observed. The impedance maxima for the different light sources differ for amplitude peak and phase changes, by 2 kΩ in amplitude and 0.5 degree in phase, respectively. In conclusion the applied UV LED illumination caused the highest increase of impedance up to approx. 6% of its dark value.

### 3.4. Light Intensity Induced PLZT Fibers Electrical Parameters Characterization and Discussion

**Figure 5.**
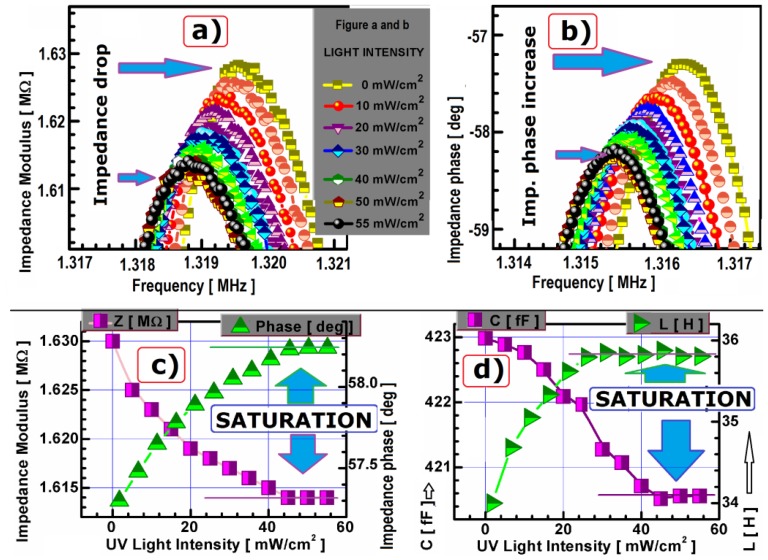
Light intensity influence on PLZT fiber impedance |Z| (**a**) and phase P (**b**) at the peak of frequency detailed presented in (**c**) and electrical equivalent circuit values for light induced variations (**d**).

The piezoelectric fibers made from PLZT powder exhibit apparent difference among characteristics proportional to UV LED illuminance (Figure 5). Surprisingly, when they are illuminated the small intensity impedance modulus peak differs markedly (2kΩ in modulus and 0.2 deg in phase for 0.5 mW/cm^2^ light change). When increasing the light intensity stepwise, the differences in impedance modulus and phase angle become smaller and finally disappear above 40 mW/cm^2^ light intensity (presented in detail in Figure 5(c) and indicated by the horizontal arrows in the Figure 5(a,b)).

In total, the difference between UV LED illuminated fiber (>40 mW/cm^2^) and unlit one is 20 kΩ in impedance modulus and 1 degree in phase (Figure 5(c)). Similar to the results described above, the same trend could be observed for the frequency. A difference of 200 Hz could be observed after switching on the light, whereas at the light intensity above 40 mW/cm^2^, no difference could be detected (Figure 5(a,b)). A shift of 1 kHz in frequency to lower values could be observed between darkness and fully illuminated fiber. The equivalent circuit elements reflected the same tendency with illumination. Namely above 40 mW/cm^2^ saturation for the capacitance C_1_, and inductance L_1_ occurred (Figure 5(d)). Parallel to the decrease of capacitance C_1_ with light illumination, the inductance L_1_ increased with characteristic saturation above 40 mW/cm^2^. The effective values of the elastic constants are light influenced, resulting in a change of the antiresonance frequency of the fiber. This effect was proved experimentally and the phenomenon was described for quartz crystals [18]. The photo excited dc electric field effect was originally quantified by means of polarization correction terms related to the elastic compliance or stiffness [19]. Our precise measurements of piezoelectric PLZT fibrous resonators also proved nonlinear change of equivalent circuit elements values. The impedance/phase maximum on the curve (at the antiresonance frequency) also depends on the magnitude of the non-linear piezoelectric stresses and strains based on the electrostriction effect and additionally on the dielectric non-linearity.

### 3.5. Light Induced Piezoelectric Parameters Evaluation

Using the resonant values above, the light driven change in piezoelectric coefficients can be calculated. Measurement of *d*_33_ piezoelectric coefficient was done using standard resonance technique (ANSI/IEEE Std 176-1987: IEEE Standard on Piezoelectricity). Resonance and antiresonance frequencies were measured together with the static capacitance $C_{33}^{T}$ of the sample (measured at 1 kHz below piezoelectric resonances). Electromechanical coupling factor *k*_33_ was calculated using the formula:

(2) $$k_{33}^{2}=\frac{\pi }{2}\frac{f_{r}}{f_{a}}\text{cotg}\left(\frac{\pi }{2}\frac{f_{r}}{f_{a}}\right)$$

elastic compliance by:

(3) $$s_{33}^{D}=\frac{1}{4f_{a}^{2}l^{2}\rho }$$

(4) $$s_{33}^{E}=\frac{s_{33}^{D}}{1-k_{33}^{2}}$$

and piezoelectric coefficient *d*_33_ by:

(5) $$d_{33}=k_{33}\sqrt{\varepsilon_{33}^{T}s_{33}^{E}}$$

where *f*_r_ and *f*_a_ are resonance and antiresonance frequencies, *ρ* is the sample density and *l* is the fiber length.

Having a light sensitive transducer application in mind, the resistance can be changed proportionally to illumination intensity by using an external UV light source. A change of 3% in elastic constants values, presented in the second and third columns of Table 2, can be observed. A difference of 4% for the piezoelectric coefficient d_33_ between dark and UV LED illuminated (with light intensity of 40 mW/cm^2^) and 10% between dark and daylight illumination can be observed for the manufactured PLZT fibers (Table 2).

To broaden the experiment measurements, UV lamp and daylight illumination on fibers were also done, but unfortunately these results can't be used for comparative analysis, because both sources emit wide range of light frequencies that were absorbed simultaneously by PLZT fibers. This effect is distinctly visible in Table 2 which shows a much more significant decrease in piezoelectric coefficients for day light than for LED for above mentioned reasons.

## 4. Conclusions

The present paper reports on the electromechanical property characterization for PLZT 7/65/35 single fiber transducers as a function of light illumination. It could be demonstrated that the properties of the fibrous elements can be tailored in three different ways by light immersion: first of all, the UV light (stimulus) can be transformed into the impedance change, secondly the piezoelectric coefficient slightly drops while the light intensity increases, and finally the amplitude characteristics shifts with the frequency.

To the authors' knowledge, the performance of the fibers is higher than for bulk PLZT (refer to [20]). This result is unexpected, because it is well known that transparent PLZT bulk ceramics are considerably better optical conductors than fibers due to the hot pressing technique used during sintering. For further investigations this would mean that the light transducer construction by the parallel fibers in transparent polymer composites could be an interesting approach for future applications. By switching on a UV LED light, a significant drop in the piezoelectric charge constant value from 228 pC/N in dark conditions to 219 pC/N was recorded. This fact definitely proves the high sensitivity of the light transducers based on fibrous form of PLZT.

## Figures and Tables

**Table 1.** The values of calcinations and sintering related coefficients for obtained PLZT fibers.

**Calcined Powder**	Mean grain size	μm	4.06
	Specific surface area	m^2^/g	5.44
	Density	g/ccm	8.07
**Sintered Fiber**	Shrinkage	%	15.5
	Porosity	%	0.51
	Grain size	μm	3.7

**Table 2.** Difference of piezoelectric coefficients and elastic constants in unilluminated (dark) and illuminated conditions for the manufactured PLZT fibers.

Parameter	*k_33_* [- ]	*s_33_^D^* [10^−12^C/N]	*s_33_^E^* [10^−12^C/N]	*d_33_* [10^−12^C/N]

PLZT 7/65/35 DARK	0.455	4.26	5.38	228
PLZT 7/65/35 UV LED	0.439	4.26	5.28	219
PLZT 7/65/35 UV LAMP	0.420	4.34	5.27	209
PLZT 7/65/35 DAY LIGHT	0.410	4.38	5.26	204
